# Computed tomography angiography in patients with active gastrointestinal bleeding*

**DOI:** 10.1590/0100-3984.2014.0014

**Published:** 2015

**Authors:** Fatima Regina Silva Reis, Patricia Prando Cardia, Giuseppe D'Ippolito

**Affiliations:** 1 Master, Professional Mastership Program in association with Medical Residency (Meparem), MD, Radiologist, Department of Imaging Diagnosis, Escola Paulista de Medicina - Universidade Federal de São Paulo (EPM-Unifesp), São Paulo, SP, Brazil.; 2 PhD, MD, Radiologist, Centro Radiológico Campinas, Hospital Vera Cruz, Campinas, SP, Brazil.; 3 Private Docent, Associate Professor, Department of Imaging Diagnosis, Escola Paulista de Medicina - Universidade Federal de São Paulo (EPM-Unifesp), São Paulo, SP, Brazil.

**Keywords:** Gastrointestinal hemorrhage, Computed tomography, Angiography, Multidetector computed tomography

## Abstract

Gastrointestinal bleeding represents a common medical emergency, with considerable morbidity and mortality rates, and a prompt diagnosis is essential for a better prognosis. In such a context, endoscopy is the main diagnostic tool; however, in cases where the gastrointestinal hemorrhage is massive, the exact bleeding site might go undetected. In addition, a trained professional is not always present to perform the procedure. In an emergency setting, optical colonoscopy presents limitations connected with the absence of bowel preparation, so most of the small bowel cannot be assessed. Scintigraphy cannot accurately demonstrate the anatomic location of the bleeding and is not available at emergency settings. The use of capsule endoscopy is inappropriate in the acute setting, particularly in the emergency department at night, and is a highly expensive method. Digital angiography, despite its high sensitivity, is invasive, presents catheterization-related risks, in addition to its low availability at emergency settings. On the other hand, computed tomography angiography is fast, widely available and minimally invasive, emerging as a promising method in the diagnostic algorithm of these patients, being capable of determining the location and cause of bleeding with high accuracy. Based on a critical literature review and on their own experience, the authors propose a computed tomography angiography protocol to assess the patient with gastrointestinal bleeding.

## Abstract (pt)

O sangramento gastrintestinal representa uma emergência médica comum, com taxas consideráveis de morbimortalidade, sendo essencial um rápido diagnóstico para se obter um prognóstico favorável. A endoscopia digestiva alta é o principal método diagnóstico, porém, em casos de sangramento maciço, o exato local de sangramento pode não ser detectável. Além disso, nem sempre está disponível um profissional treinado para a sua execução. A colonoscopia óptica na urgência tem suas limitações ligadas à ausência do preparo intestinal adequado e não permite avaliar a maior parte do intestino delgado. A cintilografia não determina a localização precisa do sangramento e não é disponível na emergência. O uso da cápsula endoscópica é inapropriado no cenário emergencial e tem custo elevado. A angiografia digital, apesar de altamente sensível, é um método invasivo, tem riscos associados ao cateterismo, além de baixa disponibilidade emergencial. Por outro lado, pela sua rapidez, ampla disponibilidade e baixa invasividade, a angiotomografia desponta como opção promissora no algoritmo diagnóstico desses pacientes, sendo capaz de determinar o local e a causa do sangramento com alta acurácia, bem como orientar o seu tratamento. Com base em revisão crítica da literatura e na nossa própria experiência, propomos um protocolo de exame de angiotomografia para o paciente com hemorragia digestiva.

## INTRODUCTION

Gastrointestinal bleeding (GIB) represents a common medical emergency, with a yearly incidence of 40-150 cases/100,000 people presenting with upper GIB and 20-27 cases/100,000 people presenting with lower GIB^(1)^, and is a common cause of admission at hospital emergency services. Among its main causes, one should highlight esophageal and gastric ulcers^(2)^. GIB is classified either as upper or lower, depending upon its origin above or below the ligament of Treitz, respectively. Approximately 75% of the patients presenting with acute GIB are upper GIB cases^(3)^. Obscure GIB is defined as bleeding with undetermined causes following upper gastrointestinal endoscopy, optical colonoscopy and small bowel radiological evaluation. It can be occult, when detected only by laboratory fecal occult blood tests, or overt, when it clinically manifests as active bleeding due to hematemesis, melena, hematochezia or enterorrhagia, frequently leading patients to seek emergency care, and possibly causing hemodynamic instability^(4)^.

In 75% of the cases, the bleeding ceases spontaneously, but recurrence is observed 25% of cases, causing mortality in 8% to 14% of cases, sometimes reaching 40% in hemodynamically unstable patients^(5)^. In addition, intermittence in GIB is frequently observed, impairing the identification of its cause. For that reason, a prompt diagnosis is essential for a favorable prognosis for such patients^(3)^. In such a context, the role played by the radiologist is that of identifying, characterizing and, whenever pertinent, treating the bleeding lesion.

## DIAGNOSTIC APPROACH STRATEGIES

The methods involved in the diagnosis of GIB include upper gastrointestinal endoscopy, optical colonoscopy, capsule endoscopy, scintigraphy, digital angiography and computed tomography (CT), most recently utilizing multiple detectors (MDCT)^(6)^.

Upper gastrointestinal endoscopy is the main diagnostic tool in upper GIB. It is a safe and widely available procedure, during which biopsies and treatment of vessels with active bleedings can be concomitantly performed, also with treatment of vessels presenting with a risk for bleeding. It has a reported sensitivity of 92-98% and specificity of 33-100%^(7-9)^. However, it is an invasive method, presenting with risk of perforations and with limited effectiveness in those cases of massive hemorrhage where large amounts of blood and presence of blood clots may impair the detection of the bleeding site^(10,11)^. In at least one series, upper gastrointestinal endoscopy was not diagnostic in 24% of cases of upper gastrointestinal hemorrhage^(12)^.

Optical colonoscopy is many times the first diagnostic method utilized in patients with lower GIB, with a sensitivity of approximately 50% for the detection of both the location and cause of the bleeding^(13)^. It is a widely accessible method, and allows for local treatment and biopsies. However, its implementation at an emergency scenario poses some obstacles such as the need for bowel preparation and the inappropriate visualization of the colon due to the presence of large amounts of blood and blood clots. Additionally, it is an invasive method, not exempt of complications such as the risk of intestinal perforation^(11,14)^. According to a comprehensive study undertaken in an emergency context, colonoscopy can identify the definite bleeding source in only 13% of the patients and the probable source in 67% of the patients^(15)^.

Capsule endoscopy, on its turn, is utilized for the evaluation of obscure bleeding, but is not feasible in an emergency scenario. Studies have demonstrated that capsule endoscopy can be diagnostic of obscure bleeding in 50-70% of the patients^(16)^. Its advantages include the capacity of screening the entire small bowel, being more sensitive in those patients presenting with clinically visible bleeding than in those presenting with occult bleeding. However, it does not allow for biopsies to be performed and, like with other endoscopic methods, a massive hemorrhage may impair the visualization of the site of active bleeding^(3,16)^.

Technetium-99m-labeled red cell scintigraphy is a noninvasive method that does not require bowel preparation and detects both arterial and venous bleeding, with a sensitivity of up to 93% and specificity up to 95%, in the presence of a bleeding rate ≥ 0.4 mL/min^(10)^. It allows for image acquisition for an extended period of time, being particularly useful in cases of intermittent bleeding. Its main limitation is the inaccurate definition of the anatomic site of the bleeding. In addition, it is a method that is not always available, requiring a long acquisition time, and difficult to be performed at an emergency unit, particularly during night shifts^(3,10)^.

Digital angiography is a widely utilized method in cases of GIB whenever upper gastrointestinal endoscopy and optical colonoscopy are negative in the identification of the bleeding site or in cases where a therapeutic intervention is required. However, it is an invasive and highly expensive method, requiring specialized professionals, a fact that may limit its availability at certain services and periods. Also, it poses risks of complications inherent to catheterization and may present false-negative results because of anatomical vascular variations. The method detects bleeding at rates ≥ 0.5 mL/min, with a sensitivity of 63-90% for upper GIB and 40-86% for lower GIB, with a specificity that may reach 100%^(3,10,17)^.

Since its clinical inception, MDCT has demonstrated high spatial and temporal resolutions^(18)^. By means of MDCT, it is possible to perform angiographic studies with multiplanar reconstructions. With that, CT angiography has become a fast, minimally invasive, and widely available method that allows for accurate localization of upper and lower GIB, particularly in the ileum and jejunum, both sites that are hardly accessible at upper digestive endoscopy and optical colonoscopy^(3,5)^. Systematic reviews evaluating the accuracy of CT angiography at GIB have demonstrated sensitivity of 85.2-89.0% and specificity of 85.0-95.0%^(17,19,20)^. A study with animal model has demonstrated that CT angiography is capable of detecting bleedings of 0.3 mL/min^(21)^.

The advantage of CT angiography over the endovascular procedures is related to its capability of accurately evaluating extraluminal abnormalities, supplying and draining vessels, regional anatomy and disease relationship with adjacent structures. Thus, the appropriate arterial mapping by means of CT angiography, before a therapeutic procedure such as digital angiography, can reduce the intervention time, exposure to radiation and the contrast agent dose, benefiting the patient^(3)^.

**Figure 1:**
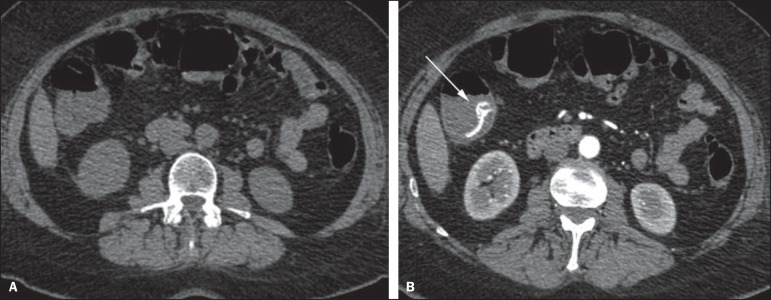
Male, 60-year-old patient. Active bleeding observed in the ascending colon, characterized by increased density with linear appearance in the arterial phase (arrow on **B**) when compared with the non-contrast-enhanced image (**A**).

The location of the bleeding and the diagnosis of its cause may play an important role in the management and treatment of such patients. For example, the accurate identification of the bleeding site can determine how the endoscopic approach will be performed, especially in those cases where it is difficult to differentiate upper from lower bleeding, with basis on the patient's clinical condition (Figure 1).

CT angiography can accurately determine the four main causes of lower GIB (angiodysplasia, colonic diverticulum, neoplasms and colitis), providing guidance for specific treatment. Thus, the location of the bleeding in the small bowel can avoid unnecessary endoscopic examination, bringing forward endovascular or surgical treatment^(10)^.

The differentiation between GIB caused by diverticulum from another caused by angiodysplasia is important, as in untreated angiodysplasia the bleeding may recur in up to 85% of the patients, contrary to diverticular disease, where 25% of the patients are affected by recurrence^(22,23)^. The tumors that cause digestive hemorrhage preferably require surgical treatment. When colectomy is performed without previous knowledge of the cause and location of the bleeding, the mortality rates reach 33% for total colectomy and 57% for partial colectomy^(24)^.

The literature describes several CT angiography protocols to evaluate GIB, with various technical variables that might influence the effectiveness of the scan^(5,13,14,18,25-40)^ (Table 1). It is not clear yet how such parameters might be combined to obtain images with high accuracy, short acquisition time and limited radiation dose, while avoiding unnecessary acquisition phases. With that in mind, the authors undertook a critical literature review in order to determine, by means of the analysis of adopted technical variables, a scan protocol that can provide the best results in the diagnosis of GIB in patients admitted to the emergency service, offering practical suggestions in order to avoid common error in the scan performance and images interpretation.

**Table 1:** CT angiography parameters for patients presenting with gastrointestinal bleeding, according to data obtained in the literature and demonstrating wide variability^(5,10,11,15,22-37)^.

Parameter	Literature
Phases	Arterial / non-contrast enhanced + arterial / non-contrast enhanced + portal / arterial + portal / non-contrast enhanced + arterial + portal / non-contrast enhanced + arterial + portal + late / arterial + enteric + portal
Oral contrast	No / yes (water)
Concentration of intravenous contrast	270-400 mg/mL
Intravenous contrast injection rate	3-5 mL/s
Intravenous contrast volume	60-160 mL
kV	100-140
mA	180-586
Thickness	0.6-5.5 mm
Number of detectors	4, 8, 16, 40, 64
Threshold (abdominal aorta)	100-150 HU

## VARIABLES INVOLVED IN THE PREPARATION AND PERFORMANCE OF CT ANGIOGRAPHY

### Preparation (fasting and oral contrast)

Fasting is not indispensable and is frequently unfeasible, as the necessity of the diagnosis originates from an emergency, and the patient might have eaten before admission. In addition, because of the intermittent nature of GIB, it is important that CT angiography be performed as soon as possible, when the active GIB is clinically detected, in order to maximize the diagnostic capability of the method^(10)^.

**Figure 2:**
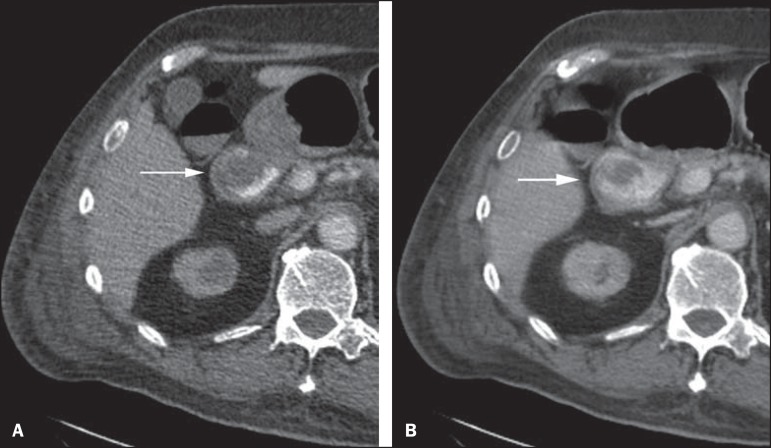
Male, 65-year-old patient presenting with hematemesis. CT angiography in the arterial phase (**A**) demonstrating active contrast medium extravasation (arrow) in the first portion of the duodenum, which enhances in the portal phase (**B**).

**Figure 3:**
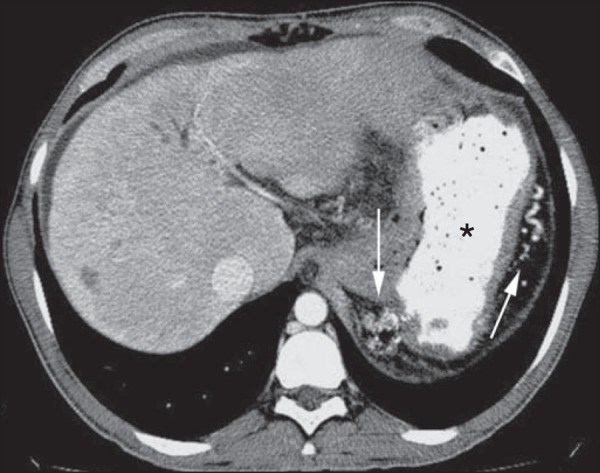
Cirrhotic patient with esophageal varices and hematemesis. In spite of extensive collateral circulation, well identified at CT (arrows), it is not possible to observe a possible active bleeding due to the presence of iodinated contrast medium in the gastric cavity (asterisk).

At CT angiography, the active bleeding is characterized by intravenous contrast medium extravasation into the bowel lumen^(6)^ (Figure 2). For that reason, the administration of neutral oral contrast medium (for example, water) or positive oral contrast material (for example, 5% iodine solution) should be avoided, as in the case of water, it can dilute the intravenously injected contrast agent upon its extravasation to the lumen of the bowel loop, thus impairing its detection; on the other hand, the positive contrast medium occupying the bowel lumen will impair the identification of the intravenously injected contrast agent extravasation, leading to a false-negative result (Figure 3). It is also possible to speed up the performance of the scan avoiding bowel preparation, thus anticipating the diagnosis and the eventually required treatment, with incontrovertible advantages for the patient^(6,41)^.

### Technical parameters (kV, mAs, slice thickness)

The reduction of radiation dose has been a constant preoccupation in the design of CT protocols, by limiting the levels of kV and mAs, besides reducing the number of CT images acquisition phases as a function of its clinical indication^(42,43)^.

In that sense, it has already been demonstrated that in the evaluation of the abdominal aorta by means of CT angiography in patients weighting less than 70 kg, the reduction of tube voltage to 90 kV maintains the diagnostic effectiveness of the method, with reduction of the radiation dose^(44)^. In another study, the authors have concluded that, as the tube voltage is reduced, the intravenous contrast volume may also be reduced without causing degradation in image quality^(45)^. However, there is no evidence in the literature that the same happens in GIB evaluation. Most articles in the literature approaching the effectiveness of CT angiography in GIB utilized tube voltage ≥ 120 kV^(5,14,18,26,29,30,32,33,37-40)^. Similarly, there are no studies demonstrating the direct impact of milliamperes magnitude and slice thickness on the effectiveness of CT angiography in the diagnosis of GIB. On the other hand, as the current literature is reviewed, the current intensity utilized in the investigation of GIB has significantly varied (Table 1), and it was not possible to establish a recommended minimum mA level. The adoption of radiation dose reduction strategies, among them the utilization of new reconstruction algorithms, such as the iterative ones (for example: ASIR^®^ or iDose^®^), should establish new milliampere standards utilized in CT scans, among those, abdominal CT angiography^(46-48)^.

### Intravenous contrast medium (concentration, dose, injection rate)

Iodine concentration, contrast agent dose and its injection rate represent variables that undoubtedly influence in some way the diagnostic quality of many CT scans^(49,50)^. However, a consensus is still to be reached in the literature about the influence of the iodine concentration in the intravenous contrast agent on the CT angiography images quality. On the other hand, better designed studies targeted on this question indicate that high density iodinated contrast media (350-400 mgI/mL) provide greater enhancement of abdominal arteries than those with lower densities (300-320 mgI/mL)^(51-53)^.

In abdominal CT, the iodine dose should be 35-45 g or approximately 1.5-2.0 mL/kg of weight (depending upon utilized iodine concentration) and, for abdominal vascular evaluation, such a dose may be reduced to 1.0-1.5 mL/kg^(54)^. Considering that, besides a merely vascular evaluation, many GIB patients require visceral evaluation, the contrast medium dose adopted by most reviewed authors is around 1.5-2.0 mL/kg (or between 100 and 150 mL) (Table 1).

It is known that a high injection rate (4-5 mL/s) is important for the acquisition of the arterial phase^(55)^, and most studies in the literature approaching GIB evaluation utilized injection rate ≥ 4 mL/s^(5,14,25,26,30-35,38,39)^.

### CT phases and acquisition times

The use of CT angiography in cases of suspicion of GIB has been implemented with a wide variability of protocols (Table 1), but a protocol comprising three acquisition phases (non-contrast-enhanced, arterial and portal phases) has most frequently been adopted^(14,25,26,28,29,31,34,35,37,39,40)^.

There is a consensus about the relevance of utilizing the non-contrast-enhanced phase in order to avoid pitfalls such as suture material, surgical clips, foreign bodies and retained contrast medium^(3,6,10)^. However, one should be careful with the utilized radiation dose, particularly in the case of young patients. For this reason, the utilization of a protocol with low radiation dose for the non-contrast-enhanced phase is recommended^(14)^.

An experimental study demonstrated that the combined utilization of the arterial and portal phases offers higher sensitivity for the detection of small bowel bleeding as compared with the utilization of a single phase^(56)^. The portal phase can improve the accuracy of the arterial phase in detecting and localizing the bleeding, particularly in cases where the bleeding originates from bowel tumors^(11)^. Another study demonstrated that the arterial phase was capable of identifying the site of the bleeding in all positive cases, and the portal phase confirmed the findings, with increased parietal enhancement and/or intraluminal accumulation of iodinated contrast medium, providing greater diagnostic reliability. On the other hand, the adoption of the delayed phase (or equilibrium phase, performed between 3 and 5 minutes after starting the injection of intravenous contrast medium) did not contribute with additional findings and improvement in the CT angiography accuracy in the evaluation of GIB patients^(33)^.

## CT ANGIOGRAPHY ANALYSIS AND IMAGING FINDINGS

**Figure 4:**
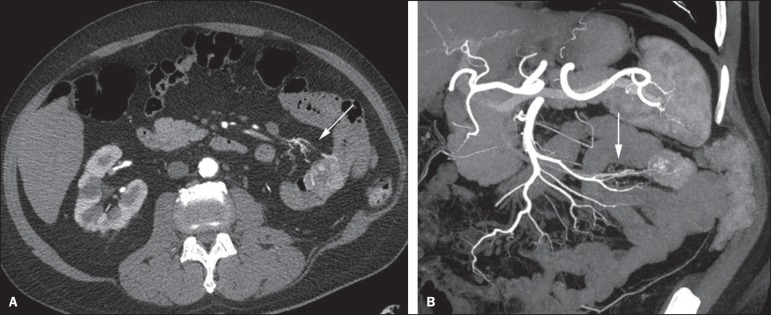
CT angiography in the arterial phase (**A**) and the corresponding angiographic MIP reconstruction (**B**). Active bleeding is observed in the jejunum (arrow on **A**). MIP reconstruction allows for identifying the branch of the superior mesenteric artery as the source of the bleeding (arrow on **B**).

**Figure 5:**
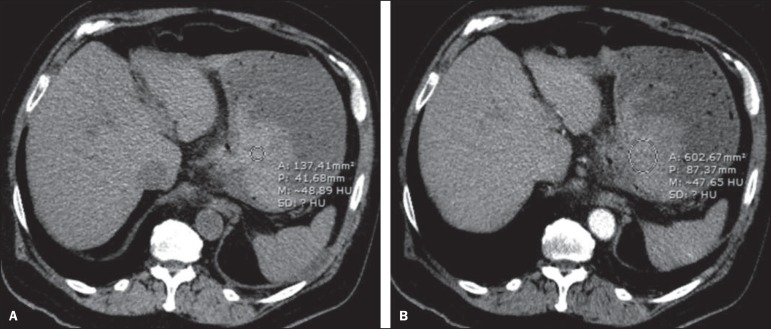
Abdominal CT angiography. Non-contrast-enhanced phase (**A**) and arterial phase (**B**). A great amount of blood is observed in the gastric chamber, characterized by hyperdense material in the non-contrast-enhanced phase (48 HU) and without enhancement in the arterial phase (47 HU). No evidence of active bleeding is observed.

**Figure 6:**
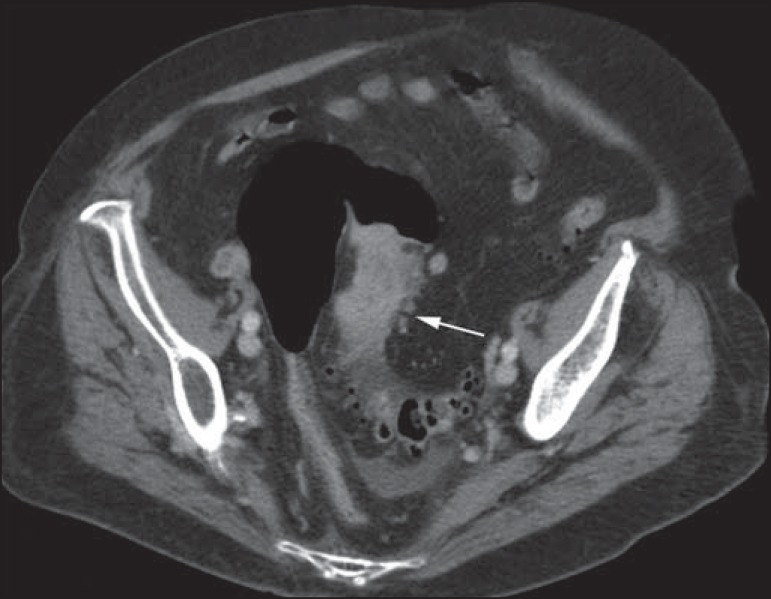
Female, 78-year-old patient presenting with enterorrhagia over the past two days. At CT angiography portal phase, a stenosing lesion is observed in the sigmoid (arrow). Additional finding: diverticular disease in the colon.

**Figure 7:**
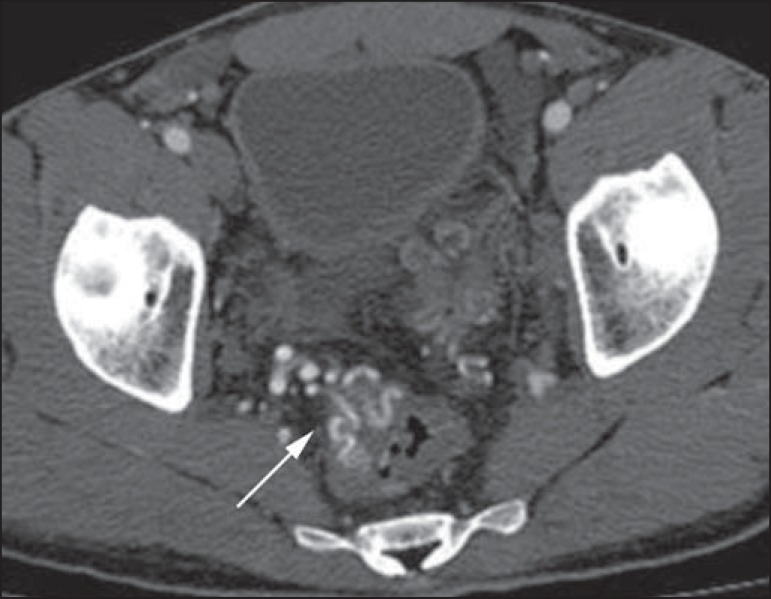
CT angiography, arterial phase in cirrhotic patient, with portal thrombosis and rectal bleeding caused by rectal and sigmoid varices (arrow).

**Figure 8:**
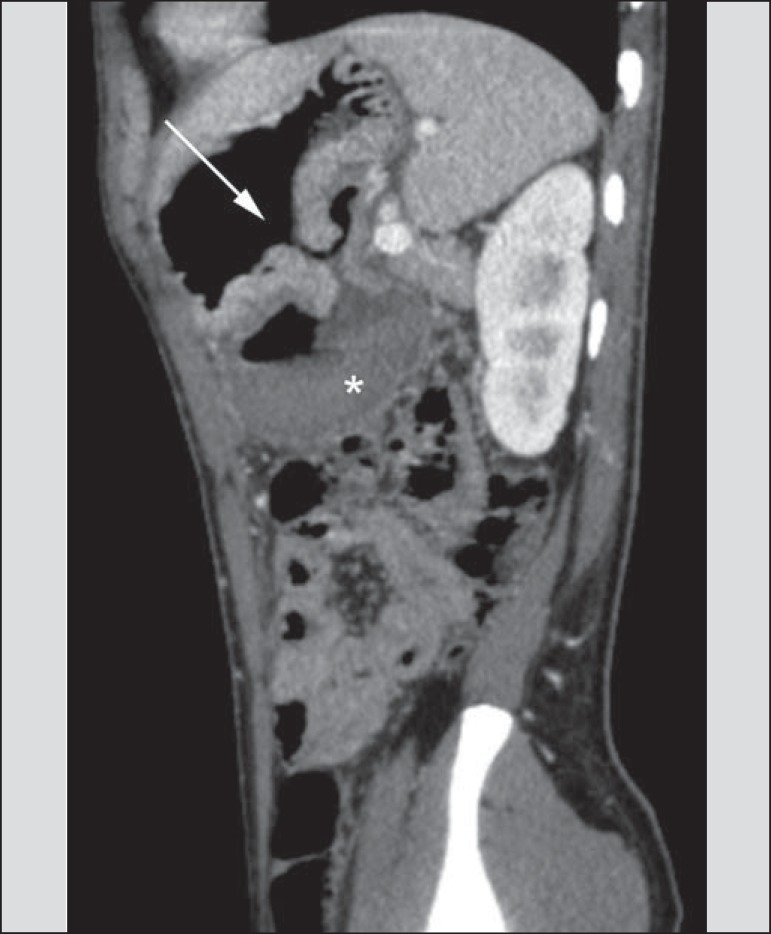
A 19-year-old hemophilia patient with abdominal pain over the past three days and hematemesis. Contrast-enhanced CT in the portal phase demonstrates the presence of a large ulcer in the greater gastric curvature (arrow) communicating the stomach lumen with retrogastric liquid collection with increased density, suggesting hematic origin (asterisk). There is no evidence of active bleeding. Upper digestive endoscopy, performed after CT, demonstrated the presence of extensive ulcerated lesion on the posterior wall of the gastric body, measuring approximately 7.0 cm, with fibrin in the base, and a large orifice (4.0 cm diameter) communicating with a large cavity, constituted of walls covered by blood and clots. Histological study of the lesion did not reveal any signs of neoplasia.

The tomographic criterion for the diagnosis of active GIB is the extravasation of the intravenously injected contrast medium to the lumen of the gastrointestinal tract (Figures 1, 2 and 4), and some authors utilize an objective density measurement > 90 Hounsfield units (HU) within the intestinal lumen^(5,29,34,35,38,40)^. In the authors' experience, as well as other authors' experience^(3,10)^, the simple visual comparison of the pre- and post-intravenous contrast images is enough to confirm the diagnosis, avoiding the already mentioned pitfalls. The contrast medium extravasation may have several appearances, namely: linear, stream, whirl, ellipsoid, or it may also occupy the entire luminal thickness of the bowel loop, and in such case one should be careful not to confuse the intraluminal extravasation of the contrast with the normal mucosal enhancement of the bowel loop, particularly in cases where it is collapsed^(3,10)^. Secondary findings should also be evaluated, and whenever present, they increase the method sensitivity. For example, acute hematoma in the non-contrast-enhanced phase indicates recent bleeding^(14)^ (Figure 5). Other findings that may indicate the cause of the bleeding and guide the management of such patients include: a) hypoenhancement and/or intestinal parietal thickening; b) presence of tumor lesion (Figure 6); c) vascular anomaly; d) esophageal varices, gastric varices or rectal varices (Figure 7); e) ulcer (Figure 8); f) abnormally enhanced polyp or diverticulum^(5,25,31,33-35,38)^.

**Figure 9:**
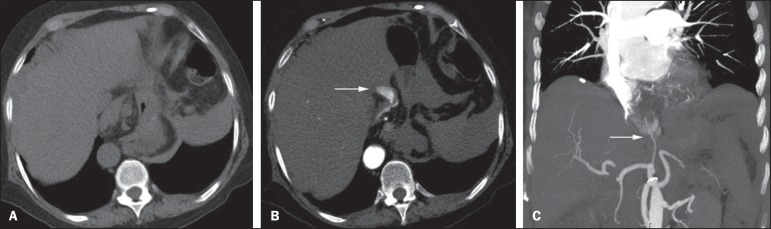
Non-contrast-enhanced CT angiography (**A**), in the arterial phase (**B**) and MIP reconstruction (**C**). Signs of active bleeding, with contrast medium extravasation (arrow on **A**) coming from the left gastric artery (arrow on **C**).

The analysis of multiplanar reformation images can enhance the diagnostic capability in acute GIB, particularly in cases of small lesions, such as angiodysplasia and arteriovenous malformations^(33)^. Reformatted coronal maximum intensity projection (MIP) images are useful in the abdominal localization of the intestinal segment with bleeding and in the evaluation of the proximal femur vascularization in cases where angiography is indicated (Figures 4 and 9). On their turn, sagittal reformatted MIP images are useful in the assessment of the rectum, as well as in the evaluation of the aorta and of the origin of the superior and lower mesenteric arteries^(10)^.

Hemorrhagic colonic diverticulosis is a frequent cause of lower GIB, commonly involving the ascending colon, while the descending colon and the sigmoid involvement is more frequently associated with inflammatory/infectious complications. Its diagnosis dispense with an accurate localization of the bleeding diverticulum, and the presence of isolated diverticulum with no evidence of active bleeding is not enough to attribute the cause of the bleeding to the diverticular disease^(57)^ (Figure 6).

In the authors' service, as tomographic images from a patient presenting with GIB, one seeks to identify the following signs: a) contrast extravasation to the gastrointestinal tract lumen; b) increase of such an extravasation in the portal phase; c) abnormal parietal enhancement (hypoenhancement), indicating ischemia or loop distress; d) intestinal wall thickening (> 3 mm); e) acute hematoma characterized by hyperattenuating area in the non-contrast-enhanced phase, either with or without enhancement after intravenous injection of the contrast medium; f) presence of tumor lesion in the gastrointestinal wall; g) signs of other GIB causes, such as vascular malformation, abnormally enhanced polyp or diverticulum and presence of ulcer.

## PROPOSAL OF CT PROTOCOL

A specific protocol for patients with a history of active GIB (Table 2) is proposed with basis on the literature review and on the authors' own experience. Initially, one should remember that, because of the clinical risk, hemodynamically unstable patients should not be submitted to CT angiography, prompting the performance of angiography or upper digestive endoscopy because of its therapeutic capability. In cases of stable patients, and considering that even in such cases it is necessary to perform CT angiography as soon as possible, the present study authors do not request patients to fast prior to the scan. Oral contrasts of any kind are not utilized either, in order not to dilute or mask possible intraluminal extravasation of the contrast medium, and to avoid delays in performing the scan. Abdominal CT angiography images are acquired in a 64-chanel Brilliance 64^®^ apparatus (Philips Medical Systems; Cleveland, OH), according to the following parameters: a) detector configuration: 64 × 0.625 mm; b) slice thickness: 1 mm; c) reconstruction thickness: 1 and 3 mm; d) 120 kV; e) mAs depending upon the automatic modulation of the radiation dose (DoseRight ACS^®^); f) pitch of 0.67; g) rotation time of 0.5 second. The acquisition time of the arterial phase is approximately 12 seconds, depending upon patient's dimensions.

**Table 2:** Proposed CT angiography protocol for abdomen and pelvis for patients presenting with active gastrointestinal bleeding. Equipment: 64-channel multidetector CT apparatus.

Parameter	Proposed
Phases	Non-contrast enhanced + arterial + portal
Oral contrast	No
Intravenous contrast concentration	350 mg/mL
Intravenous contrast injection rate	4 mL/s
Intravenous contrast volume	100 mL or 1.5 mL/kg
kV	120
mA	Automatic
Thickness	1 mm
Threshold (abdominal aorta)	100 HU
Time for arterial phase initiation	20 seconds after threshold
Time for portal phase initiation	40 seconds after threshold

Considering the relevance of the rational utilization of radiation, the non-contrast-enhanced phase is acquired with a low-dose protocol. A volume of 120 mL of non-ionic iodinated contrast medium is intravenously injected, preferably into the antecubital vein, by means of an automated Injektron 82 CT/DG^®^ (Guerbet) injection pump at a rate of 4 mL/s, followed by 30 mL saline solution flush, at the same injection rate. Arterial and portal phases acquisition is performed as they are complementary and, as previously mentioned, increase the overall effectiveness of the scan. The beginning of the arterial phase acquisition is defined by means of the automated bolus triggering technique that defines a circular region of interest within the abdominal aorta, immediately above the celiac trunk. The arterial phase initiates 20 seconds after a density of 100 HU is achieved in the descending aorta. The portal phase initiates 40 seconds after such a peak. Once a fixed time is established, one may adopt 40- and 70-second spans after starting the intravenous contrast injection, in order to obtain the arterial and portal phases respectively. One should consider that delaying the arterial acquisition time is less detrimental than anticipating it, as a longer interval allows for the contrast medium to cross the thin vascular network of the intestinal wall and reach its lumen, in cases of active bleeding. However, it is important to observe that better results are obtained when the automated bolus tracking program is utilized.

**Figure 10:**
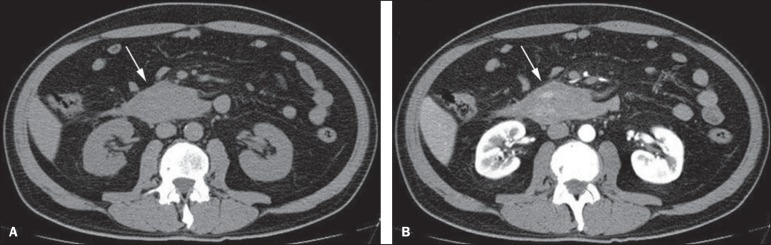
Posterior wall ulcer in the third portion of the duodenum with signs of active bleeding characterized by contrast medium extravasation identified by difference in density between non-contrast-enhanced phase (**A**) and contrast-enhanced phase (**B**) (arrows) and by the enhancement appearance.

The images analysis initiates by the non-contrast-enhanced phase, by seeking to identify spontaneously hyperattenuating spots in the intestinal lumen that might represent pitfalls in the contrast-enhanced phases and the presence of high density areas on the intestinal wall suggesting the diagnosis of acute hematoma (Figure 10). Subsequently, such images are carefully compared with those obtained in the arterial and portal phases, seeking to identify areas of contrast medium extravasation in the gastrointestinal lumen (Figure 10) and secondary signs as the above mentioned ones. Finally, by means of multiplanar angiographic reconstructions with the MIP technique, one seeks to identify with greater accuracy not only the origin of the bleeding, but also, whenever possible, the supplying vessel ( Figures 4 and 9) and possible vascular anatomic variants. Such data may be useful in the planning and performance of therapeutic angiography.

## CONCLUSION

GIB is a frequent medical emergency, with important morbimortality rates, requiring a fast diagnostic tool capable of localizing the site and cause of the bleeding, and consequently allowing for the institution of appropriate treatment as soon as possible. Abdominal CT angiography is a fast, minimally invasive and widely available method that can precisely and accurately determine the location and cause of digestive hemorrhage. The effectiveness of the method is optimized with the implementation of a scan protocol that takes into consideration the several technical variables that somehow influence the detection of the bleeding site without neglecting factors that might limit the radiation dose. Based on such considerations, the present study authors suggest that for hemodynamically stable patients presenting with active GIB, CT angiography be performed as first-line diagnostic tool as soon as possible, in order to maximize the bleeding detection capability and assist in the therapeutic planning.
